# Dynamics and complexity of body temperature in preterm infants nursed in incubators

**DOI:** 10.1371/journal.pone.0176670

**Published:** 2017-04-27

**Authors:** Kerstin Jost, Isabelle Pramana, Edgar Delgado-Eckert, Nitin Kumar, Alexandre N. Datta, Urs Frey, Sven M. Schulzke

**Affiliations:** 1 Department of Biomedical Engineering; University of Basel, Basel, Switzerland; 2 Department of Neonatology, University of Basel Children’s Hospital (UKBB), Basel, Switzerland; 3 Computational Physiology and Biostatistics, University of Basel Children’s Hospital (UKBB), Basel, Switzerland; 4 Department of Pediatrics, University of Basel Children’s Hospital (UKBB), Basel, Switzerland; University of Illinois at Urbana-Champaign, UNITED STATES

## Abstract

**Background:**

Poor control of body temperature is associated with mortality and major morbidity in preterm infants. We aimed to quantify its dynamics and complexity to evaluate whether indices from fluctuation analyses of temperature time series obtained within the first five days of life are associated with gestational age (GA) and body size at birth, and presence and severity of typical comorbidities of preterm birth.

**Methods:**

We recorded 3h-time series of body temperature using a skin electrode in incubator-nursed preterm infants. We calculated mean and coefficient of variation of body temperature, scaling exponent alpha (T_alpha_) derived from detrended fluctuation analysis, and sample entropy (T_SampEn_) of temperature fluctuations. Data were analysed by multilevel multivariable linear regression.

**Results:**

Data of satisfactory technical quality were obtained from 285/357 measurements (80%) in 73/90 infants (81%) with a mean (range) GA of 30.1 (24.0–34.0) weeks. We found a positive association of T_alpha_ with increasing levels of respiratory support after adjusting for GA and birth weight z-score (p<0.001; R^2^ = 0.38).

**Conclusion:**

Dynamics and complexity of body temperature in incubator-nursed preterm infants show considerable associations with GA and respiratory morbidity. T_alpha_ may be a useful marker of autonomic maturity and severity of disease in preterm infants.

## Introduction

Poor control of body temperature, i.e., the inability to maintain a normal body temperature by balancing heat loss and heat production in a feedback loop over a wide range of environmental temperatures, is a significant clinical problem in preterm infants and associated with mortality and major morbidity in this population [1–3]. The autonomic nervous system controls several important effector systems of thermoregulation in neonates including brown adipose tissue, vasomotor tone, and sweat glands [4]. In preterm infants assessed during the first few days of life, those systems are characterized by autonomic dysfunction, e.g., by poor vasomotor response to cold stress [5, 6]. It is widely accepted that a stable thermal environment is crucial for preterm infants as lowest mortality and morbidity in infants born before 33 weeks gestational age (GA) have been shown for an admission temperature ranging from 36.5 to 37.2°C [3]. Thermal stability in newborn infants, defined by the World Health Organization as a state where the core temperature lies between 36.8 and 37.3°C [7], has beneficial effects on other autonomic processes such as control of breathing and heart rate, and supports growth and sleep regulation [8, 9]. Control of body temperature is particularly important during the physiologically challenging post partum transition and the first few days of life, as environmental conditions and temperatures may change rapidly in this period of time. In order to treat autonomic dysregulation of body temperature, preterm infants are typically nursed in incubators that allow for servo-controlled adjustment of the incubator’s air temperature based on the difference between the infant’s body temperature and a preset target temperature.

Time series of biological signals such as body temperature often reveal weak statistical long-range correlation properties which are an expression of internal ordering of the time series and a result of internal regulation of the biological system [10]. Temporal dynamics and statistical complexity of time series of fluctuating physiological signals can be quantified using mathematical techniques such as detrended fluctuation analysis (DFA) and sample entropy. Scaling exponent alpha derived from DFA describes the self-similarity (scaling) of a biological signal in a time series across a range of sizes of time windows. Sample entropy reflects the amount of irregularity of a time series by quantifying to what extent a pattern is likely to reoccur in the time series. These methods are established mathematical tools to quantify statistical long-range correlations in time series and have been successfully used for monitoring of disease severity and risk prediction [10–15]. DFA has been successfully used to quantify long-range correlations of body temperature in healthy term infants beyond the neonatal age [16]. In these infants, DFA indicates an association between statistical long-range correlations of body temperature and postnatal age. To the best of our knowledge, abovementioned techniques have not been applied to study temperature dynamics and complexity in preterm infants nursed in incubators, where body temperature is tightly regulated and of utmost clinical importance. In critically ill adults, a decrease in temperature curve complexity i.e., increased statistical regularity in a time series of temperature values, is a significant predictor of mortality [17]. While the predictive value of temperature curve complexity has not been established in preterm infants, recent data suggest that assessing complexity of autonomic dysregulation in terms of heart rate variability using sample entropy, i.e., complexity in an autonomic system closely related to thermoregulation, is a promising approach for predicting sepsis and sepsis-associated mortality in preterm infants [18, 19]. Consequently, analysis of dynamics and complexity of body temperature in preterm infants might reveal objective, clinically relevant information on presence and development of autonomic dysregulation and potential associations with patient characteristics and typical comorbidities of preterm birth.

We aimed to quantify dynamics and complexity of body temperature in very preterm (<32 weeks GA) and/or very low birth weight (<1500 g BW) infants nursed in incubators using descriptive statistics and time-series analyses. We hypothesized that dynamics and complexity of body temperature quantified as mean (T_mean_), coefficient of variation (T_cv_), scaling exponent alpha derived from DFA (T_alpha_), and sample entropy (T_SampEn_) of body temperature measured over the first five days of life are associated with demographic characteristics of the infants (GA, body size and body proportions at birth, sex), presence and severity of typical comorbidities of preterm birth, and behavioural state and extent of handling during the measurement.

## Methods

### Design

We conducted a prospective, observational single center study in the neonatal intensive care unit at the University of Basel Children’s Hospital, Switzerland. The study was approved by the local ethics committee and written informed consent was obtained from all parents within the first 24 h after birth and prior to the start of the measurements.

### Patients

We included very preterm (<32+0 weeks GA) and/or very low birth weight (<1500 g BW) infants. Exclusion criteria were major congenital malformations, asphyxia, or infants in whom treatment was directed towards palliative care.

### Measurements

We recorded time series of body temperature measured over a period of 3 consecutive hours at a sampling frequency of 0.1 Hz in incubator-nursed preterm infants. Measurements were started each morning at 8.30 am on each of the infant’s first five days of life. Thus, measurements allowed for collection of >1000 data points and effects of circadian rhythm on outcomes were minimized. Body temperature was measured with a skin electrode (Skin Temperature Probe, Weyer Medical Products, Kuerten Germany; Range: 0–50°C; max. measurement error +/- 0.1°C) that was attached to the trunk with an adhesive and positioned between infant and mattress. Depending on the infant’s position, the sensor was placed between abdomen and mattress (prone position) or between back and mattress (supine position). This non-invasive setup, i.e., ‘the zero heat flux method’, has been shown to result in a consistent and reliable approximation of body core temperature [20, 21]. The incubator (Thermocare Vita Weyer GmbH, Kuerten, Germany) was set to servo-control mode using a target body temperature between 36.8 and 37.0°C. No manual adjustment of air temperature or target body temperature was performed during the 3-h measurements.

### Data acquisition

Time series of temperature data were extracted from the RS232 port of the incubator’s temperature control unit at a sampling frequency of 0.1 Hz and stored on a mobile computer using HyperTerminal software (Hilgraeve, Inc., Monroe, MI, USA).

Synchronized video recordings (Microsoft 1080 HD Sensor (Life Webcam)) were obtained and manually scored by trained researchers at an identical frequency of 0.1 Hz to determine the infant’s behavioural state (awake; quiet sleep; active sleep; assessment based on facial expression, breathing pattern and activity level). This type of analysis was validated in previous studies and allows for assessing sleep without EEG recording in preterm babies [22]. Additionally, positioning of the infant and the extent of handling (open incubator doors in % of recording time and doors-perturbance score, calculated as the cumulative product of time and number of open incubator doors) were documented. On average, video scoring of a 3-h measurement was completed within 1 h.

### Data processing, data analysis and quality control

Temperature data were graphically plotted and visually checked using custom-written scripts developed in Matlab (The MathWorks, Inc., Natick, MA, USA). Measurement time intervals during which the temperature sensor was misplaced or other technical artifacts occured were excluded from the analysis. The amount of excluded data within each measurement was included in the sensitivity analysis of outcomes (for details, see below). We calculated outcome parameters using the statistical software R [23] and the R-package zoo [24]. The time series analysis parameters were calculated using our own implementations in R (occasionally accessing C libraries to reduce run time) of well-known algorithms [25–27]. Many of the implementations are based on the TISEAN package [26].

### Outcome parameters

Predefined outcome parameters included descriptive statistics (mean (T_mean_) and coefficient of variation (T_cv_) of body temperature) over the measurement period, the scaling exponent alpha (T_alpha_) as determined by detrended fluctuation analysis (DFA), and measures of sample entropy (T_SampEn_). T_alpha_ values represent type and degree of correlation of temperature values in the given time series, with higher T_alpha_ values indicating stronger long-range correlations [28], i.e., stronger temperature control. T_SampEn_ reflects complexity, i.e., ‘non-regularity’ or ‘non-predictability’ of the temperature time series data. Typically, SampEn values decrease from baseline in association with disease [17].

### Detrended fluctuation analysis (DFA)

To estimate the long-range correlation properties in body temperature, we analysed 0.1 Hz-time series of body temperature using DFA. The scaling exponent alpha obtained via DFA was calculated using a geometric window increase with exponent equal to 1.5 and no overlap of windows. The minimal time window size was set to 100 seconds to exclude parts dominated by discretization errors, as the temperature measurements were obtained with an accuracy of only two places after the decimal point. For any given window size, a minimum of 15 windows was required for the calculations. This requirement determined the size of the maximal window size considered, which therefore varied from time series to time series. The data were linearly detrended. The algorithm of DFA used is described in [29] and [26]. DFA plots were visually inspected. Such plots display the logarithm of the root-mean-square errors with respect to the local trend within a given time window versus the logarithm of the corresponding window size. Those plots in which, by visual inspection, a non-linear relationship between the logarithm of the root-mean-square error and the logarithm of the window sizes was suspected were excluded from further analysis. In other words, the scaling exponents derived from such measurements were not included in the regression analysis. In total, 6/285 (2%) of measurements were discarded based on visual inspection of DFA plots.

### Sample entropy (T_SampEn_)

The sample entropy was calculated on coarse-grained time series constructed on the basis of collapsing the original values within a window of the size of the scale of interest to one value, namely the average of the measurements over the length of the window. The scale considered (size of window) was 6, which, at a sampling frequency of 0.1 Hz, corresponds to a time window of 1 minute. The sample entropy was calculated using a comparison length of m = 2 points, and a tolerance of *r = 0*.*2* *sd, where sd stands for the standard deviation of all temperature measurements, according to the algorithm described by Richman and Moorman [25] and in the citations therein.

### Sensitivity analysis

We performed sensitivity analyses if parts of a measurement had to be excluded due to technical problems (e.g., detached temperature sensor). Therefore, outcomes calculated from all technically acceptable parts of the measurement were compared to those calculated from the single longest, technically acceptable part of the measurement in order to evaluate whether the amount of missing values introduced in the data cleaning process influences results.

### Statistical analysis

Aiming at 80% statistical power on the 5% significance level, we aimed to recruit a total of n = 90 infants in order to analyse a minimum of n = 76 preterm infants (expected loss to follow-up 15% due to technical reasons and potential withdrawal of parental consent) allowing for linear regression analysis of at least three continuous independent predictor variables of medium effect size (f^2^ = 0.15) [30, 31].

Baseline demographics of study participants were extracted from medical records. We performed linear regression analyses to assess associations between outcomes (T_mean_, T_cv_, T_alpha_, T_SampEn_) and potential predictors. Considered predictors included demographics (GA, BW, BW z-score, relative weight loss over the first five days of life (%), sex); relevant comorbidities associated with preterm birth (early onset sepsis (EOS) (no; suspected (defined as histologically approved chorioamnionitis or CRP>20 mg/mL and antibiotic treatment >5 days), proven (criteria for suspected early onset sepsis plus positive blood cultures)), germinal matrix-intraventricular haemorrhage (maximum grade 1 to 4) documented according to Papile [32]), positioning (prone, supine) during the measurement, phototherapy due to hyperbilirubinaemia, level of respiratory support at study (none, nasal positive pressure support with and without intermittent increase in nasal flow, mechanical ventilation), time to last caffeine dose (hours) at start of the measurement (all infants received caffeine)), and the infant’s behavioural state and handling characteristics (see above).

We first used univariable, multilevel modelling to allow for clustering on the individual level given that repeated measurements over the first five days of life were analysed (one 3-h measurement per day in each patient). This step included exploring associations of all considered predictors with outcomes and a p-value < 0.1 was considered to indicate potential relevance of a predictor. We then built multivariable, multilevel linear regression models for each outcome and did stepwise backward elimination of predictors that were not significantly associated with the outcome (p<0.05 considered statistically significant). We defined a best model depending on the coefficient of determination of the model (R^2^) and compared models using the likelihood ratio test. Models were explored for interaction of predictors and model diagnostics included plotting of residuals against fitted values. We log-transformed outcomes that were not normally distributed (T_cv_, T_SampEn_). Statistical analysis was performed using Stata software (StataCorp. 2009. Stata Statistical Software: Release 11. College Station, TX: StataCorp LP).

## Results

**Fig 1:**
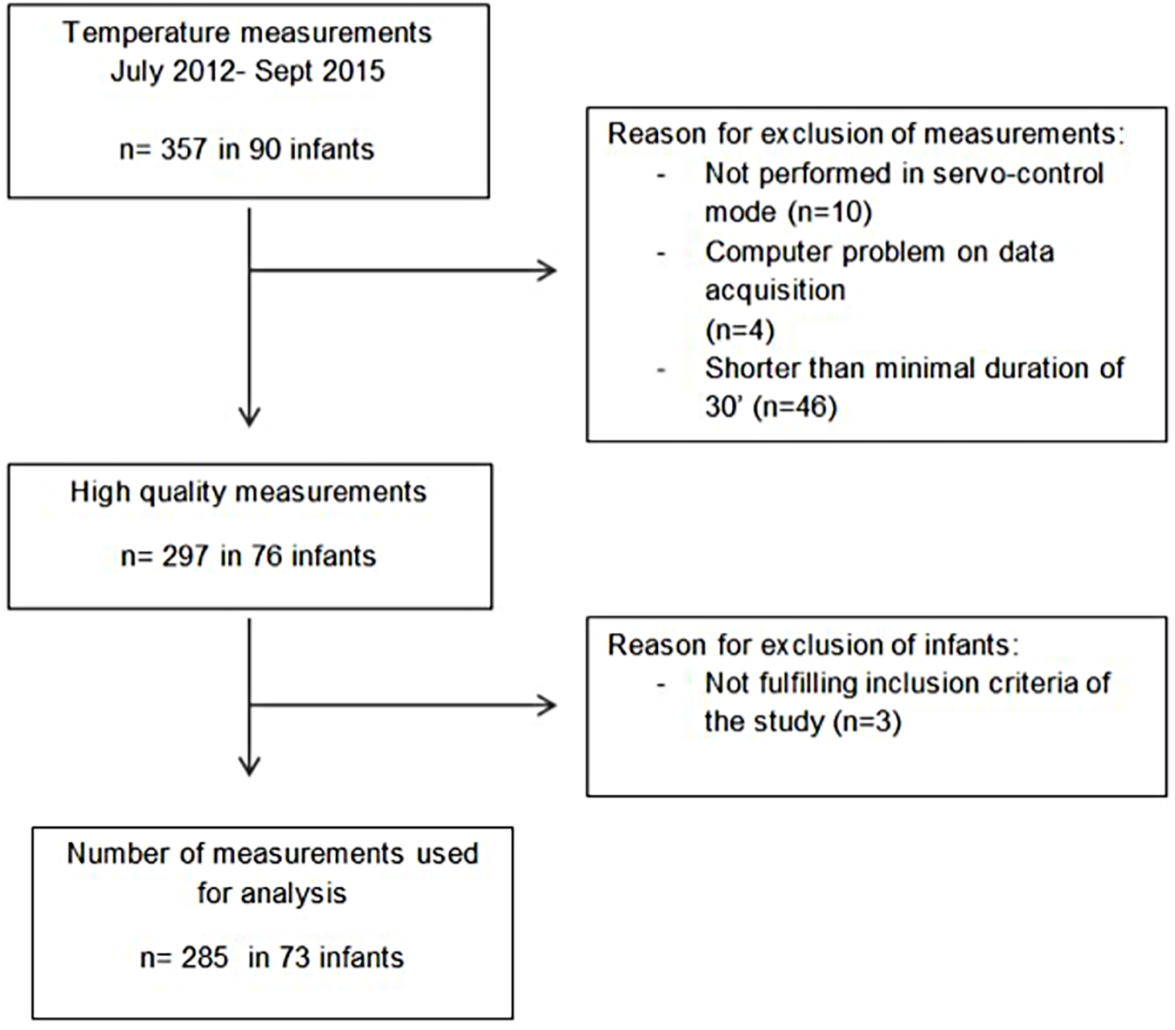
Flow of patients and measurements through the phases of the study.

Between July 2012 and September 2015, a total of 357 temperature measurements, each recorded over a period of 3 consecutive hours at a sampling frequency of 0.1 Hz were conducted in 90 infants. Data of satisfactory technical quality (see methods) were obtained from 285 measurements (79.8%) in 73 infants (81.1%). Fig 1 shows the flow of patients through the phases of the study. Study participants had a mean (range) GA of 30.1 (24.0–34.0) weeks and a mean (range) birth weight (BW) of 1244 (490–1900) g. Baseline characteristics of participants are summarized in Table 1.

**Table 1:** Baseline characteristics of study participants.

Variable	
Participants, n (% male)	73 (64)
Gestational age at birth (weeks)	30.05 (2.46)
Birth weight (g)	1244 (337)
Birth weight z-score	-0.94 (1.07)
Weight loss in first week of life (%)	2.35 (4.10)
Maximum level of respiratory support, n measurements (%) • None • Continuous positive airway pressure • Endotracheal ventilation	• 108 (38) • 161 (56) • 16 (6)
*Early onset sepsis, n infants (%) • None • Suspected • Proven	• 43 (59) • 19 (26) • 11 (15)
**Germinal matrix/intraventricular haemorrhage, n infants (%) • None • Grade 1 • Grade 2 • Grade 3 • Grade 4	• 63 (86) • 5 (7) • 0 (0) • 4 (6) • 1 (1)

Data are presented as mean (standard deviation) unless stated otherwise.

*Defined as no; suspected (CRP>20mg/ml, antibiotic treatment >5 days, histologically proven chorioamnionitis); proven (suspected plus positive blood cultures).

**Maximum grade (1 to 4) documented according to Papile classification [32].

### Association of outcomes with considered predictors

Results of univariable, multilevel linear regression analyses of all outcomes are summarized in Tables 2 and 3.

**Table 2:** Univariable, multilevel linear regression analyses of mean and coefficient of variation of body temperature.

	T_mean_				T_cv_^a^			
Variable	Coeff beta	p-value	95% CI	R^2^ between; overall	Coeff beta	p-value	95% CI	R^2^ between; overall
GA (weeks)	0.012	0.183	-0.006 to 0.031	NA	-0.016	0.249	-0.044 to 0.011	NA
Weight (g)	<0.001	0.975	<-0.001 to <0.001	NA	<0.001	0.146	<0.001 to <0.001	NA
BW z-score	-0.034	0.117	-0.076 to 0.008	NA	-0.018	0.587	-0.084 to 0.048	NA
Weight loss (%)	<0.001	0.876	-0.005 to 0.006	NA	0.002	0.796	-0.011 to 0.014	NA
Male gender	-0.012	0.805	-0.219 to 0.032	NA	0.029	0.704	-0.119 to 0.177	NA
EOS	-0.115	0.081	-0.244 to 0.014	7; 1	0.172	0.096	-0.030 to 0.374	6; 2
IVH	-0.123	0.186	-0.306 to <-0.059	NA	0.063	0.673	-0.358 to 0.231	NA
Respiratory support	0.070	0.380	-0.087 to 0.227	NA	0.407	0.011	0.095 to 0.719	11%; 2%
Prone position	0.007	0.860	-0.072 to 0.086	NA	-0.114	0.199	-0.364 to 0.076	8%; 1%
Phototherapy	0.132	<0.001	0.084 to 0.179	8%; 10%	0.250	0.001	0.102 to 0.398	8%; 4%
Time to last caffeine (h)	<-0.001	0.749	-0.005 to 0.004	NA	<-0.001	0.855	-0.011 to 0.009	NA
Duration (min)	-0.001	0.024	-0.002 to <-0.001	7%; 3%	<-0.001	0.824	-0.003 to 0.002	NA
Perturbance	0.082	0.342	-0.087 to 0.250	NA	0.359	0.207	-0.198 to 0.916	NA
Time awake (%)	<-0.001	0.401	-0.002 to 0.001	NA	-0.005	0.017	-0.009 to <-0,001	2%; 3%
Time active (%)	<-0.001	0.521	-0.002 to 0.001	NA	-0.005	0.031	-0.010 to -0.001	6%; 3%
Duration SC (min)	0.001	0.122	<-0.001 to 0.003	NA	0.009	0.001	0.004 to 0.014	16%; 6%

^a^Log-transformed to achieve normal distribution; GA: Gestational age; Weight: Weight at study; BW z-score: Birth weight z-score; Weight loss: % difference of birth weight and weight at study; EOS: Early onset sepsis (none; suspected; proven); IVH: Germinal matrix-intraventricular haemorrhage (none, grade 1–2 vs. grade 3–4); Respiratory support at study (none; continuous positive airway pressure; endotracheal ventilation); Phototherapy: Phototherapy during measurement due to hyperbilirubinaemia; Time to last caffeine: Time from last caffeine dose measured at start of observation (h); Duration: Duration of measurement (min); Perturbance: Perturbance score, i.e., number of open incubator doors x time of open incubator doors during measurement; Time awake: % of measurement spent awake; Time active: % of measurement spent awake or in active sleep; Duration SC: Average duration (min) of sleep cycles (reappearance of active sleep) within one measurement.

**Table 3:** Univariable, multilevel linear regression analyses of scaling exponent alpha and sample entropy of body temperature.

	T_alpha_				T_SampEn_^a^			
	Coeff beta	p-value	95% CI	R^2^ between; overall	Coeff beta	p-value	95% CI	R^2^ between; overall
GA (weeks)	-0.026	<0.001	-0.036 to -0.015	23%; 9%	0.054	0.007	0.015 to 0.094	9%; 3%
Weight (g)	<-0.001	<0.001	<-0.001 to <-0.001	30%; 14%	<0.001	<0.001	<0.001 to <0.001	16%; 5%
BW z-score	-0.017	0.254	-0.046 to 0.012	NA	0.058	0.250	-0.041 to 0.157	NA
Weight loss (%)	-0.002	0.506	-0.006 to 0.003	NA	0.004	0.621	-0.013 to 0.022	NA
Male gender	<-0.001	0.982	-0.067 to 0.065	NA	-0.102	0.349	-0.319 to 0.113	NA
EOS	0.189	<0.001	0.114 to 0.264	29%; 11%	-0.487	0.001	-0.769 to -0.204	15%; 4%
IVH	-0.044	0.500	-0.170 to 0.083	NA	0.399	0.065	-0.025 to 0.822	NA
Respiratory support	0.274	<0.001	0.161 to 0.387	29%; 14%	-0.759	0.001	-1.192 to -0.327	15%; 5%
Prone position	-0.072	0.072	-0.151 to 0.006	9%; 2%	0.211	0.167	-0.089 to 0.510	NA
Phototherapy	0.066	0.011	0.015 to 0.117	12%; 3%	-0.265	0.009	-0.463 to -0.067	2%; 3%
Time to last caffeine (h)	-0.003	0.074	-0.007 to <0.001	6%; 2%	0.009	0.227	-0.005 to 0.023	NA
Duration (min)	<0.001	0.320	<-0.001 to 0.001	NA	<0.001	0.949	-0.003 to 0.004	NA
Perturbance	0.107	0.276	-0.085 to 0.299	NA	-1.035	0.005	-1.765 to -0.307	4%; 4%
Time awake (%)	-0.001	0.074	-0.003 to <0.001	7%; 3%	0.004	0.180	-0.002 to 0.010	NA
Time active (%)	<0.001	0.989	-0.002 to 0.002	NA	0.005	0.210	-0.003 to 0.012	NA
Duration SC (min)	0.003	0.001	0.001 to 0.005	3%; 7%	-0.012	<0.001	-0.018 to -0.005	11%; 8%

Please refer to legend of Table 2.

### Mean temperature (T_mean_) and coefficient of variation (T_cv_)

There was no association of T_mean_ and T_cv_ with demographic characteristics of study participants. In univariable analysis, T_mean_ was negatively associated with early onset sepsis (EOS) and germinal matrix/intraventricular haemorrhage (IVH), and positively associated with phototherapy and duration of the measurement (Table 2). The best multivariable model included EOS, phototherapy, and duration of the measurement and explained 27% of the variation in T_mean_ between infants (p<0.001, R^2^ = 0.27) (Table 4). T_cv_ was negatively associated with EOS, time awake and time active, and positively associated with respiratory support, phototherapy and duration of sleep cycles (Table 2). The best multivariable regression model included only phototherapy and duration of sleep cycles (Table 4). This model explained 19% of the variability in T_cv_ between infants (p<0.001, R^2^ = 0.19).

**Table 4:** Multivariable, multilevel linear regression analyses of all outcomes.

	Coeff beta	p-value	95% CI	R^2^ between; overall
T_mean_				27%; 16%
EOS (proven)	-0.139	0.019	-0.256 to -0.023	
Phototherapy	0.141	<0.001	0.092 to 0.191	
Duration (min)	-0.002	0.001	-0.002 to -0.001	
T_cv_^a^				19%; 9%
Phototherapy	0.359	0.020	0.056 to 0.661	
Duration SC	0.007	0.013	0.001 to 0.012	
T_alpha_				38%; 19%
GA	-0.016	0.021	-0.029 to -0.002	
BW z-score	-0.044	<0.001	-0.069 to -0.020	
Respiratory support	0.205	0.003	0.071 to 0.339	
T_SampEn_^a^				15%; 7%
GA	0.065	0.002	0.023 to 0.106	
BW z-score	0.104	0.039	0.005 to 0.202	
Phototherapy	-0.218	0.031	-0.416 to -0.019	

^a^Log-transformed to achieve normal distribution; GA: Gestational age; BW z-score: Birth weight z-score; EOS: early onset sepsis (none; suspected; proven); respiratory support at study (none; continuous positive airway pressure; endotracheal ventilation); Phototherapy: Phototherapy during measurement due to hyperbilirubinaemia; Duration: Duration of measurement (min); Duration SC: Average duration (min) of sleep cycles (reappearance of active sleep) within one measurement.

### Scaling exponent alpha derived from DFA (T_alpha_)

**Fig 2:**
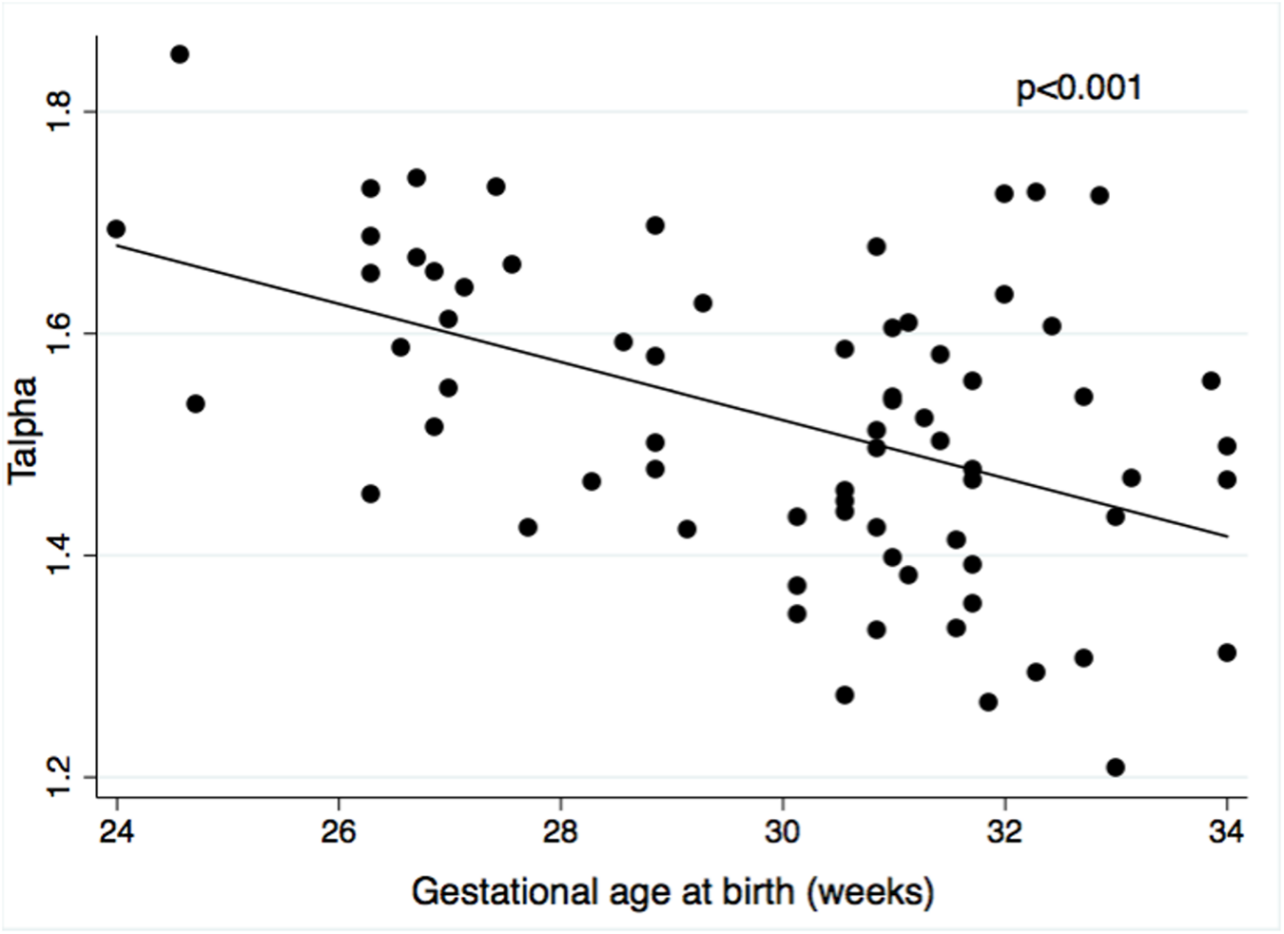
Scaling exponent alpha (T_alpha_) over gestational age (GA). Average T_alpha_ over the first five days of life derived from detrended fluctuation analysis of temperature time series plotted against GA at birth. Multilevel linear regression analysis demonstrated a significant negative association between T_alpha_ and GA at birth (p<0.001, R^2^ = 0.29).

**Fig 3:**
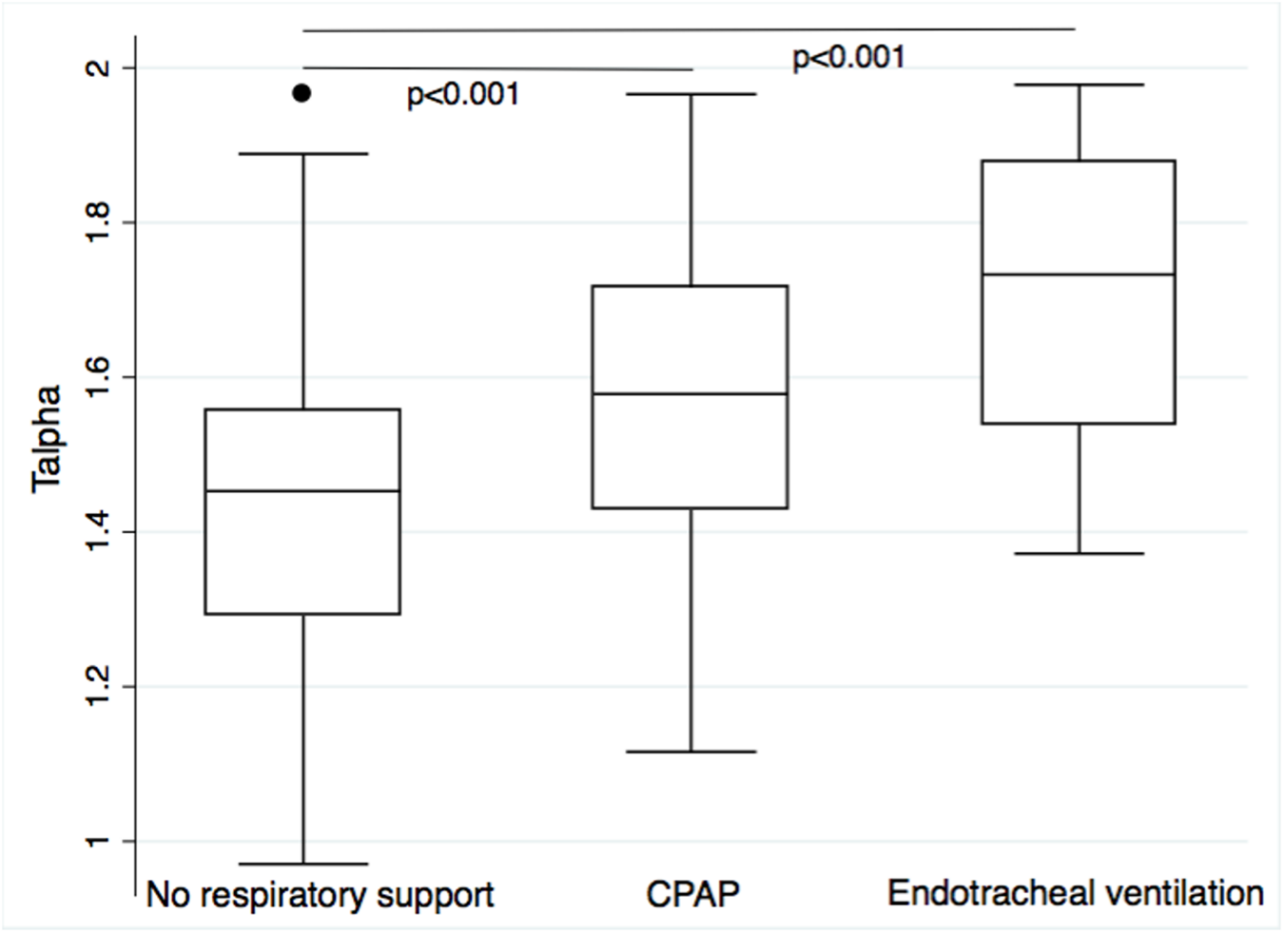
Scaling exponent alpha (T_alpha_) over respiratory support. T_alpha_ grouped by the level of respiratory support present during a temperature measurement. Multilevel linear regression analysis demonstrated a significant positive association between T_alpha_ and stepwise increase of respiratory support from none to continuous positive airway pressure (CPAP), and from CPAP to endotracheal ventilation (p < 0.001, R^2^ = 0.29).

**Fig 4:**
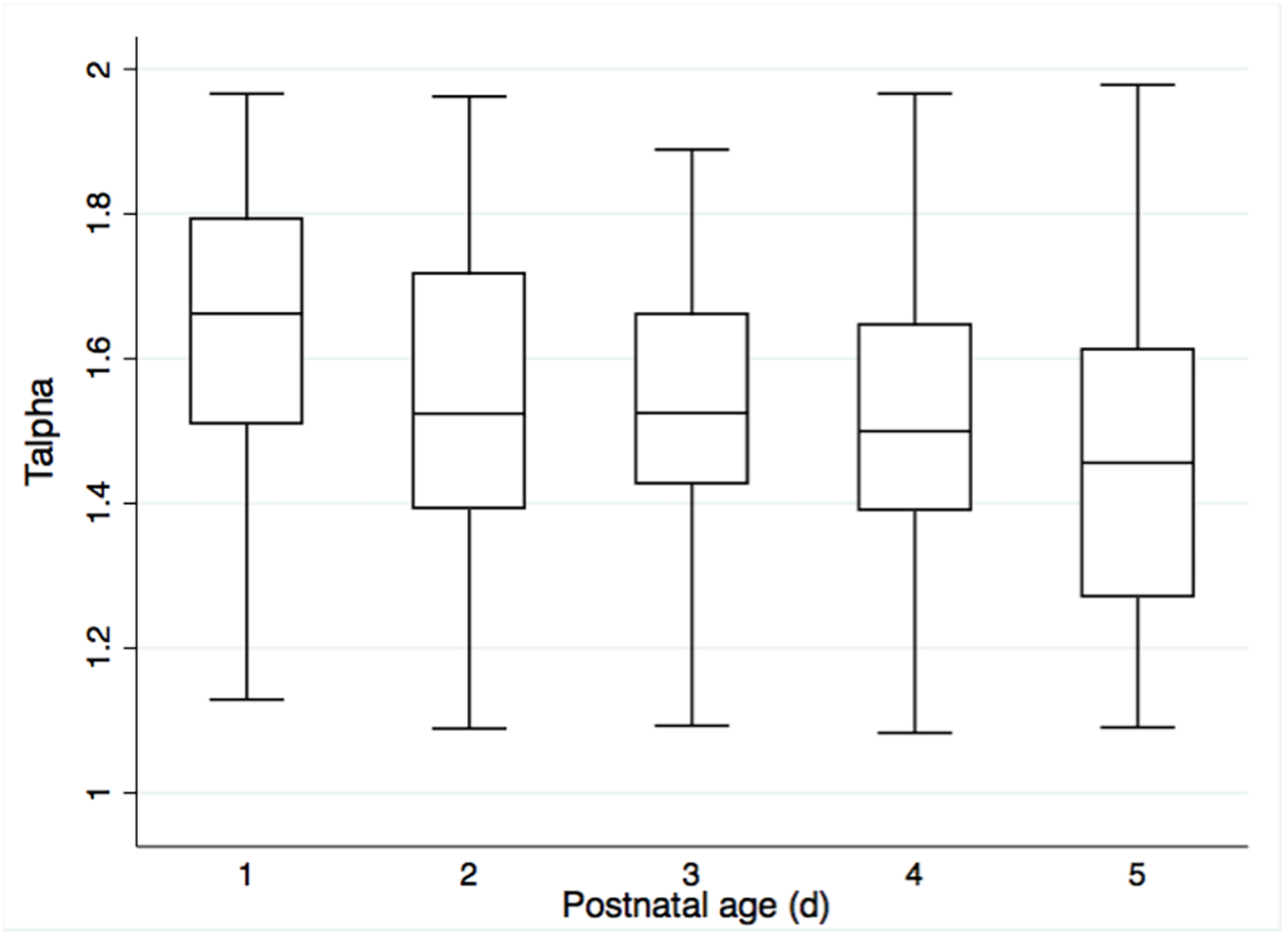
Scaling exponent alpha (T_alpha_) over postnatal age. T_alpha_ decreased from a mean (SD) of 1.67 (0.18) on day one of life to 1.46 (0.22) on day five of life (p<0.001).

Univariable linear regression analysis suggested a negative association of T_alpha_ with GA and BW, a positive association of T_alpha_ with several comorbidities (EOS, level of respiratory support, phototherapy) and a negative association of T_alpha_ with prone position, time to last caffeine dose and time awake (Table 3). The best multivariable, multilevel model confirmed significant negative associations of T_alpha_ with GA and BW z-score and the positive association of T_alpha_ with the level of respiratory support (p<0.001, R^2^ = 0.38) (Table 4). On average, T_alpha_ decreased by 0.03 (95% CI: -0.04 to -0.02; p<0.001) per week of GA at birth (Fig 2). A change in the level of respiratory support corresponded to a stepwise increase in T_alpha_ (from no support to continuous positive airway pressure: T_alpha_ +0.13 (95%CI: +0.08 to +0.18; p<0.001); from continuous positive airway pressure to endotracheal ventilation: T_alpha_ +0.27 (95% CI: +0.16 to +0.39; p<0.001)) (Fig 3). In terms of longitudinal changes of T_alpha_ over the first five days of life, there was a negative association of T_alpha_ with postnatal age of study participants (Fig 4). The mean (SD) difference of T_alpha_ between first and fifth day of life was 0.14 (0.30) (p<0.001).

### Sample entropy (T_SampEn_)

In univariable regression analysis, T_SampEn_ was positively associated with demographic characteristics (GA and BW) and negatively associated with several comorbidities (EOS, level of respiratory support, phototherapy) and measurement conditions (perturbance score, duration of sleep cycles) (Table 3). Multivariable, multilevel regression established a model including significant predictors GA, BW z-score and phototherapy (p<0.001, R^2^ = 0.15%) (Table 4): There was a mean increase of T_SampEn_ of 0.07% (95% CI: 0.02 to 0.11%; p = 0.002) per week of GA and of 0.1% (95% CI: 0.01 to 0.2%; p = 0.039) per unit of BW z-score. Use of phototherapy resulted in a mean decrease of T_SampEn_ of -0.2% (95% CI: -0.4 to -0.02%).

### Sensitivity analysis

In 70/285 (25%) of measurements, a reason for partial exclusion of temperature data occurred (e.g., detachment of temperature sensor as verified by synchronized video analysis). The mean (SD) duration of excluded parts from a 3-h measurement period was 37 (26) min. Table 5 shows a comparison of outcome parameters using all technically acceptable parts vs. only using the single longest continuous, technically acceptable part of the measurements; T_cv_ and T_SampEn_ but not T_mean_ were significantly different (paired t-test). However, results of regression analyses were essentially identical in terms of associations and effect sizes of predictors. Thus, results reported in this manuscript refer to analyses done using all technically acceptable parts of a measurement.

**Table 5:** Sensitivity analysis related to amount and fragmentation of data.

Variable	All acceptable parts	Single longest acceptable part	Mean diff (95%CI); p-value^b^
T_mean_	37.0 (0.3)	37.0 (0.3)	0.0 (-0.02 to 0.02); p = 0.863
T_cv_^a^	-66 (57)	-102 (61)	36 (-116 to -81); p<0.001
T_SampEn_^a^	-1.58 (0.70)	-1.38 (0.76)	-0.20 (-0.35 to -0.06); p = 0.007

Data are presented as mean (standard deviation)

^a^Log-transformed to achieve normal distribution

^b^paired *t*-test; CV: coefficient of variation; T_SampEn_: Sample entropy; Mean Diff: Difference of outcome comparing all technically acceptable parts vs. only the single longest technically acceptable part.

## Discussion

We found that dynamics and complexity of body temperature are quantifiable in incubator-nursed preterm infants with a success rate of about 80%. Daily measurements during the first five days of life under servo-controlled conditions indicated that T_mean_ and T_cv_ are independent of GA, BW, and sex and are only modestly associated with typical comorbidities of preterm birth and behavioural state of the infants. Fractal long-range correlations of body temperature characterized by T_alpha_ were significantly associated with demographics (GA, BW z-score) and severity of respiratory disease as measured by the level of respiratory support. The effect size of those associations was considerable (R^2^ = 0.38) and T_alpha_ decreased over the first few days of life. Complexity of body temperature in terms of T_SampEn_ was only modestly associated with demographics (GA, BW z-score) and presence of phototherapy (R^2^ = 0.15). The main results of this study were not affected by behavioural state and extent of handling of infants and were robust to sensitivity analyses considering amount and fragmentation of temperature data within a 3 h-measurement.

### Comparison of findings with previous literature

Maintaining body temperature within a narrow target range is challenging for preterm infants within the first week of life given their relatively high body surface to weight ratio, large metabolic cost of maintaining temperature balance, and immature temperature control systems [33–35]. Reassuringly, we found that T_mean_ of infants in our study was within the target range of 36.8 to 37.0°C independent of the degree of prematurity and despite a small but significant effect of comorbidities such as EOS, IVH and use of phototherapy. These results confirm findings from previous studies indicating that servo-controlled temperature care based on skin temperature of preterm infants is safe in the clinical routine setting of a neonatal intensive care unit [21, 33–40].

To our best knowledge, this is the first study to assess fractal long-range correlations of body temperature in preterm infants. Stern et al. previously showed that mean (SD) T_alpha_ obtained from monthly temperature measurements in healthy term infants increased from 1.42 (0.07) at 4 weeks to 1.58 (0.04) at 20 weeks of age [16]. They interpreted this to be due to a maturational effect towards a more deterministic system with increasing age post term. In contrast, our data showed a decrease in mean (SD) T_alpha_ from 1.67 (0.18) on day one of life to 1.46 (0.22) on day five of life. However, there are important methodological differences between our study and that of Stern et al. including population characteristics and study setting (preterm infants nursed in incubators shortly after birth vs. healthy term infants assessed at home several weeks to months after birth) and data acquisition (different timing, frequency, duration, and sampling rate of temperature measurements) These differences in methodology might explain the discrepancy in the direction of the association between T_alpha_ and postnatal age.

We found lower complexity, i.e., higher T_alpha_ and lower T_SampEn_, to be associated with the degree of prematurity and growth restriction at birth, and with several comorbidities of preterm infants during their first days of life. These are novel observations suggesting that lower complexity of temperature time series in preterm infants can be interpreted as less deterministic temperature control associated with prematurity, growth restriction, and severity of respiratory disease. In fact, an increase in T_alpha_ was associated with a stepwise increase in the level of respiratory support required during the first few days of life after adjusting for prematurity and intrauterine growth restriction, i.e., increasing loss of complexity reflected the degree of disease in a ‘dose-response’ relationship. Varela et al. showed that a decrease in temperature curve complexity in 50 critically ill adults with multi organ failure was associated with a higher mortality rate [17]. They found significantly higher T_alpha_ and lower T_SampEn_ in hourly temperature data, summarized as lower complexity, in patients who did not survive their intensive care unit stay after adjusting for age. Hence, and similar to our findings, these authors interpreted the loss of complexity in temperature fluctuations as a marker of severity of disease and, additionally, as an indicator of poor prognosis.

### Strengths and limitations of the study

Strengths of this study include assessing a population at high risk of autonomic dysregulation of body temperature in whom thermal balance is of critical importance; using a safe, non-invasive, and reliable method of acquiring temperature time series data including synchronized video recordings to adjust for behavioural state, sleep and handling of the infant; a sample size very close to that projected in pre-emptive power calculations; and sensitivity analyses to test the robustness of results in relation to potential issues due to missing values in time series data. Limitations of our study include the inability to provide exact timing of comorbidities such as IVH, the lack of evaluating the complete temperature feedback loop including air temperature and heat output of the incubator and the fact that we did not collect longitudinal data allowing for assessment of substantial maturational changes in temperature control over weeks to months. Such data would allow for a more complete understanding of the physiological mechanisms underlying temperature fluctuations and provide a setup to study the predictive value of long-range correlations of body temperature in incubator-nursed preterm infants for medium-term outcomes such as growth or resolution of autonomic dysregulation. However, this study provides novel methods and biologically important information on quantitative characterization of temperature regulation in preterm infants in a temperature-wise very controlled environment.

### Clinical relevance and future applications

Quantifying the dynamics and complexity of body temperature from time series is a first step towards better understanding of the immature autonomic thermal control system and its interaction with the degree of prematurity and typical morbidities of preterm infants. A key criterion for discharge of preterm infants from the nursery is their ability to maintain body temperature upon transfer from an incubator to an open cot. Arguably, future real-time analysis of temperature time series in a longitudinal fashion might allow for quantitative assessment of maturation of the temperature control system and provide a rational basis for transfer of infants from an incubator to an open cot. Currently, this decision is primarily based on body weight of infants [41, 42], however, there is little evidence to suggest a specific body weight indicating readiness for transfer, i.e., such transfers are typically a matter of trial and error despite the fact that thermo-neutrality is important for adequate weight gain and has beneficial effects on other autonomic control systems such as breathing and heart rate [9, 43, 44].

## Conclusion

Dynamics and complexity of body temperature in preterm infants nursed in incubators can be quantified and show associations of considerable effect size with the degree of maturity at birth, intrauterine growth, and respiratory morbidity. Behavioural state, handling of infants, and missing data have limited impact on fractal temperature dynamics. T_alpha_ is a promising, observer-independent, and robust tool for assessing autonomic maturation and severity of disease in preterm infants. Longitudinal studies are required to evaluate whether T_alpha_ is useful for predicting clinically relevant outcomes and resolution of autonomic dysregulation.
